# A New Deal for Global Health R&D? The Recommendations of the Consultative Expert Working Group on Research and Development (CEWG)

**DOI:** 10.1371/journal.pmed.1001219

**Published:** 2012-05-15

**Authors:** John-Arne Røttingen, Claudia Chamas

**Affiliations:** 1 Department of Health Management and Health Economics, Institute for Health and Society, University of Oslo, Oslo, Norway; 2 Harvard Kennedy School, Harvard University, Cambridge, Massachusetts, United States of America; 3 Centre for Technological Development in Health, Fiocruz, Rio de Janeiro, Brazil

## Abstract

John-Arne Røttingen and Claudia Chamas, chairs of the the Consultative Expert Working Group on Research and Development (CEWG), summarize their recent report recommending to the World Health Assembly that a global health R&D treaty be developed.

In May 2012 at the World Health Assembly, member states of the World Health Organization (WHO) have the opportunity to make substantial progress on a major global health challenge: how to catalyse new knowledge for diseases that primarily affect the global poor and for which patents provide insufficient market incentives. The existing system can neither adequately develop nor deliver health technologies addressing health concerns mainly or only constituting a problem in developing countries. Those markets have no ability to pay the high prices needed to recover research and development (R&D) costs, which is the way the current system operates. We need mechanisms that delink the cost of R&D from the price of products.

There have been many positive efforts to tackle these issues, including the establishment of diverse public–private product development partnerships (PDPs) like the Medicines for Malaria Venture and Drugs for Neglected Diseases initiative. But these efforts have only treated the symptoms exhibited by the system failures and not the root causes. WHO has been the arena for discussion and analysis of these issues for 10 years including the work of the Commission on Intellectual Property Rights, Innovation and Public Health and the agreement on a Global Strategy and Plan of Action on Public Health, Innovation and Intellectual Property [1],[2]. But sustainable solutions for access to medicines have not been established. More than a hundred proposals have been put forward by submissions from different stakeholders during these processes and to follow on expert groups, but the approaches are often fragmented and sometimes competing. Last month the Consultative Expert Working Group on Research and Development (CEWG), which we have had the honour of chairing, published a comprehensive analysis concluding with a set of bold recommendations for the WHA to consider [3].

Acting upon the CEWG recommendations would constitute a transformative change rather than an incremental improvement. We do call for more money—this is not new in global health, but even more importantly we call for money that is used in smarter ways. We propose new strategies for how research in this area can be conducted and a new paradigm for how financial contributions should be determined based on the concept that both the costs and benefits of R&D should be shared. We recommend a role for WHO in the stronger coordination of R&D and suggest pooling of financial investments to secure efficient allocations to where demands and opportunities are identified through active participation of developing countries.

We recommend the creation of a system complementary to the existing intellectual property regime in which patents currently constitute the main incentive for investment. We call for R&D conducted within an *open knowledge innovation* framework where knowledge is either in the public domain or free to use through appropriate licensing arrangements. Such “open” approaches have been used successfully in other sectors and foster innovation. This will allow for products to be delivered at competitive prices that developing countries can afford. We also recommend more extensive use of patent pools to better share knowledge, and to use prizes as an incentive mechanism for discovering new products.

Today, developing countries must rely on their own limited financial and human resources to develop technologies they need, or they must depend on aid or technology transfers based on charity or political decisions. Neither the self-sufficiency nor the charitable approaches are sustainable. Our proposal constitutes a third way forward where these R&D investments are treated like the global public goods they are such that all countries—rich and poor, developed and developing alike—contribute according to the size of their economy. This conceptualization of R&D investments also underscores that it is inefficient to generate knowledge that cannot be fully exploited and that all countries should pay their share of this global resource. Based on analysis of current needs and investments, we are recommending countries allocate at least 0.01% of GDP to this global public good, which would result in a doubling of current investments.

The major challenge for new policies is not to get agreement on them, but to make them acted upon and fully implemented. There is a debate on the value of soft law versus hard law approaches to making globally agreed upon policies deliver. Some argue that the only way to get agreement on strong and specific enough measures is through soft laws, since hard laws often end with watered down commitments, and that soft law thereby can achieve more. However, the reason for soft laws to be more easily agreed upon is exactly that they are less committing. This line of argument then ends with two options: soft laws with clear commitments but which are not committed to, or hard laws with weak commitments that are committed to. Both options can end with poor policy outcomes. Our firm belief is that it is time to break this Catch-22. The CEWG proposes that our set of recommendations should be combined in a legally binding instrument with clear commitments; a new international convention.

So far, the only existing international health laws adopted under the WHO Constitution are the Framework Convention on Tobacco Control and the International Health Regulations. These can be said to be regulating “global public *bads*” in that they either reduce sales and consumption of a harmful product or reduce the transmission of infectious agents. It is time to also regulate the commitments needed to produce “global public *goods*”. We should learn from the environmental sector where the Multilateral Fund to protect the ozone layer and the new Green Climate Fund have been established through conventions. We believe that a convention on global health R&D meets the necessary criteria for when international health law is an appropriate instrument [4]. It is a way to secure a systemic and sustainable solution since it creates a formalized platform for the future where countries can be held accountable.

We know that our recommendations will be met by both applause and scepticism. Systemic changes will always be difficult since many interests are at stake. This is why the status quo is so resilient. However, the millions of potentially preventable deaths each year demand changes. We must be willing to endure a phase of disequilibrium [5]. This requires leadership and we hope WHO can provide such adaptive leadership. We believe our report will be a good platform for member states of WHO to enter into negotiations to establish a convention for global health R&D. They must not be caught up in time wasting and exhausting processes. Now is the time.

## Abbreviations

CEWG: Consultative Expert Working Group on Research and Development
PDP: product development partnership
R&D: research and development
WHO: World Health Organization
